# Effect of Enriching Gingerbread Cookies with Elder (*Sambucus nigra* L.) Products on Their Phenolic Composition, Antioxidant and Anti-Glycation Properties, and Sensory Acceptance

**DOI:** 10.3390/ijms24021493

**Published:** 2023-01-12

**Authors:** Patrycja Topka, Szymon Poliński, Tomasz Sawicki, Aleksandra Szydłowska-Czerniak, Małgorzata Tańska

**Affiliations:** 1 Department of Food Plant Chemistry and Processing, Faculty of Food Sciences, University of Warmia and Mazury in Olsztyn, 10-718 Olsztyn, Poland; 2 Confectionery Factory “Kopernik” S.A., 87-100 Toruń, Poland; 3 Department of Analytical Chemistry and Applied Spectroscopy, Faculty of Chemistry, Nicolaus Copernicus University in Toruń, 87-100 Toruń, Poland; 4 Department of Human Nutrition, Faculty of Food Sciences, University of Warmia and Mazury in Olsztyn, 10-718 Olsztyn, Poland

**Keywords:** functional food, antioxidant capacity, phenolic acids, flavonoids, advanced glycation end products, sensory analysis

## Abstract

Elder products are still underutilized sources of phytochemicals, mainly polyphenols, with extensive pharmacological effects on the human body. In this study, gingerbread cookies covered in chocolate (GC) were enriched with elderflower dry extract (EF) and juice concentrate (EB). The cookies (GC, GCEF, and GCEFEB) and the additives (EF and EB) were analyzed for total phenolic content (TPC), phenolic compound profile, antioxidant capacity (AC), and advanced glycation end products’ (AGEs) formation in both the free and bound phenolic fractions. Sensory analysis of the cookies was performed using an effective acceptance test (9-point hedonic scale), and purchase intent was evaluated using a 5-point scale. It was found that the flavonoid content was significantly increased (20–60%) when EF and EB were added to the cookies. Moreover, the EF addition to chocolate-covered GCs enhanced the content of phenolic acids (up to 28%) in the bound phenolic fraction. An increase in the AC values of enriched cookies was found, and the free phenolic fraction differed significantly in this regard. However, inhibition of AGEs by elder products was only observed in the bound phenolic fraction. In addition, EF and EB improved the overall acceptance of the cookies, mostly their taste and texture. Thus, elder products appear to be valuable additives to gingerbread cookies, providing good sensory quality and functional food characteristics.

## 1. Introduction

The production of gingerbread in Poland has a long tradition, with the first mention dating back to the 14th century. The Polish city of Toruń is particularly related to the tradition of its baking; it was there that the first gingerbread factory in Poland was established in 1751. Gingerbread is a sweet bakery product that can take the form of a cake or a cookie, in which the key ingredients are specified spices. The formula for its dough may vary depending on local custom, but there are some characteristic ingredients used for cake baking, such as wheat flour, rye flour, honey, cinnamon, and ginger. In mass production, honey is increasingly being replaced by invert sugar or glucose-fructose syrup. This confectionery product belongs to the category of bakery products with a long shelf life [1,2,3].

The presence of a relatively large amount of spices in gingerbread makes it a value-added product with health-promoting properties. Recent studies confirmed that the consumption of spices typically used in gingerbread production, such as cinnamon, ginger, clove, nutmeg, and coriander, has protective effects for disease management, including cardiovascular ailments, diabetes, hypertension, dyslipidemia, stroke, cataracts, macular degeneration, impaired cognition, inflammation, and cancer [4,5,6,7,8,9]. These diseases belong to the group of aging diseases, as their incidence increases significantly with age, and these diseases contribute to high rates of morbidity and mortality in the elderly. Many bioactive compounds with pharmacological effects on the human body have been identified in gingerbread spices, primarily phenolic compounds and essential oils. For example, cinnamaldehyde, cinnamyl acetate, cineole, coumarin, ethyl cinnamate, linalool, humulene, β-caryophyllene, and τ-cadinol were determined in cinnamon bark; 6-gingerol, 6-paradol, 6-gingerdiol, gingerdione, shogoal, zingiberene, citral, bisabolene, cineol, α-farnesene, β-phellandrene, and zingerone in ginger rhizome; carvacrol, thymol, eugenol, cinnamaldehyde, and eugenyl acetate in clove; eugenol, myristicin, elemicin, sabinene, safrole, methyl eugenol, α-pinene, β-pinene, myristic acid, and 4-terpineol in nutmeg; linalool, geraniol, geranyl acetate, and camphor in coriander [10,11,12,13,14].

Due to consumer preferences, a significant portion of gingerbread products available on the market are coated in dark chocolate, which is a source of flavanols, including epicatechin, catechins, and procyanidins [15,16,17,18]. Additionally, the gingerbread can be enriched with other pro-health ingredients, e.g., rose hip pulp powder [19], camel thorn and peppermint powders [20], sea buckthorn flour [21], blended roasted pumpkin, blended roasted beetroots, and baked tomatoes [22]. Nevertheless, there are no published studies that consider the incorporation of black elder (*Sambucus nigra* L.) products in gingerbread. Literature data showed that elderberries (the fruits of the elder tree) are rich in many bioactive compounds (mainly anthocyanins, including cyanidin 3-glucoside and cyanidin 3-sambubioside, phenolic acids, flavonols and flavonol esters, lectins, and vitamin C) that exhibit diverse health functions, including antioxidant, anti-inflammatory, anticancer, anti-influenza, antimicrobial, and antidiabetic, as well as having neuroprotective activities and being cardiovascular protective [23,24,25]. In addition, elderflowers (the flowers of the elder tree) are a rich source of flavonoids such as kaempferol, astragalin, quercetin, quercetin-3-*O*-glucoside, rutin, isoquercitrin, and hyperoside. Moreover, pectins, tannins, and phenolic acids have also been found in elderflowers [23,26]. Elderflowers can be used both for the prevention and therapy of a wide array of diseases due to their immunomodulatory, anti-inflammatory, antimicrobial, antioxidant, and antiviral activities [27,28]. 

Although the phytochemicals in elder products have complex polypharmacological actions, the most popular of these is their antioxidant effect. A recent study showed that the antioxidant activity of elderflower and elderberry extracts resulted from the presence of phenolic compounds, mainly flavonols, phenolic acids, and anthocyanins [29,30,31]. Furthermore, more and more researchers highlight the potential anti-aging mechanisms of polyphenols, including antioxidant signaling, preventing cellular senescence, targeting microRNA, influencing NO bioavailability, and promoting mitochondrial function [32,33,34,35,36]. Huang et al. [37] also reported the anti-glycation activity of elderberry phenolics. Advanced glycation end products (AGEs), also known as glycotoxins, are created in products through a nonenzymatic reaction between reducing sugars and free amino groups of proteins, lipids, or nucleic acids, especially after exposure to higher temperatures and lower moisture. Excessively high levels of AGEs have been linked to the development of diabetes and several other chronic diseases [38,39]. Although the use of single polyphenols as pharmaceuticals in the prevention of age-related disorders is gaining popularity, the consumption of polyphenol-rich foods appears to be more physiologically relevant in daily life and can probably provide more benefits due to the cumulative effect of multiple polyphenols.

Taking into account the health-promoting properties of spices, dark chocolate, and elder products, it is possible to put forward the hypothesis that gingerbread cookies coated in dark chocolate and incorporated with elderflower extract and elderberry juice could be a product that positively affects consumers’ health. 

Therefore, this work aimed to quantify, for the first time, the antioxidant and anti-glycation properties of gingerbread cookies with the commercially available elder products, elderflower dry extract (EF) and elderberry juice concentrate (EB). In the study, the free (extracted with 80% methanol) and bound (hydrolyzed with 2 M sodium hydroxide) phenolic fractions were analyzed separately to show the effect of elder products on the bioavailability of phenolic compounds for the human body. It is widely recognized that the sensory experience is important for the acceptance of a new product; therefore, a sensory analysis of the studied cookies was also performed.

## 2. Results and Discussion

### 2.1. Phenolic Composition and Antioxidant Properties of Elderflower Dry Extract and Elderberry Juice Concentrate Used for Gingerbread Cookie Fortification

The characteristics of free and bound phenolic fractions in elderflower dry extract (EF) and elderberry juice concentrate (EB) used for gingerbread cookie fortification are shown in Table 1. 

There were significant differences in the total content of phenolic compounds (TPC) between EF and EB for both free and bound phenolic fractions. It is noteworthy that the free phenolic fraction content in EF (831.14 μg/g) was nearly two times higher than that in EB (437.69 μg/g). Even greater differences were observed in the content of the bound phenolic fraction between EF (850.94 μg/g) and EB (90.58 μg/g). These results confirm that EF was a richer source of free and bound phenolics than EB. In our previous study, a water extract from elderflowers also revealed approximately 2.5 times higher TPC (81.9 mg gallic (GA) acid/g) than a water extract from elderberries (TPC = 32.5 mg GA/g) [40]. Additionally, flowers of different elderberry species or interspecific hybrids (TPC = 7410–40137 μg GA/g) contained significantly higher levels of phenolic compounds compared to berries (TPC = 2687.6–6831.1 μg GA/g) [41].

Six phenolic acids were identified in the studied extracts (Table 1). Benzoic acid was the main phenolic acid in the free phenolic fraction of EF extract (49%), while ferulic acid was the most abundant in the bound phenolic fraction of EF (91%). Ferulic acid was also the main phenolic acid in the free phenolic fraction of EB extracts (84%), while *p*-coumaric acid was the main phenolic acid in the bound phenolic fraction of EB extract (47%). The sum of phenolic acids in the free phenolic fraction of EF (54.84 μg/g) was 8.8 times lower than the sum of phenolic acids in the bound phenolic fraction of EF (484.50 μg/g). In contrast, the sum of phenolic acids in the free phenolic fraction of EB (176.89 μg/g) was 4.5 times higher than the sum of phenolic acids in the bound phenolic fraction of EB (39.78 μg/g). Furthermore, fourteen flavonoids were identified in the studied extracts. In both the free and bound phenolic fractions of EF and also in the free phenolic fraction of EB, the main identified flavonoid was quercetin-3-*O*-glucoside (which accounted for 60%, 55%, and 66%, respectively), while epicatechin was the most abundant in the bound phenolic fraction of EB (which accounted for 71%). It should be emphasized that the sum of flavonoids in EF, both in free (219.07 μg/g) and bound (109.63 μg/g) phenolic fractions, was significantly higher than in EB, in which the content of these compounds was 148.20 μg/g and 45.12 μg/g, respectively.

Comparing the two raw materials used as gingerbread ingredients, it can be seen that EF can be a better source of phenolic compounds than EB. However, in the case of EB, larger amounts of free phenolic acids may be incorporated into the cookies. Furthermore, a large proportion of the flavonoids in EF were bound. Although both elder products were high in ferulic acid and quercetin-3-*O*-glucoside, EF also contained other phenolic acids (caffeic, benzoic and hydroxybenzoic acids) and flavonoids (isorhamnetin, kaempferol, myricetin and quercetin derivatives), while EB was high in benzoic and *p*-coumaric acids, as well as epicatechin and quercetin. Other studies also showed that hydroxycinnamic acids represented the major share of phenolics in elderberries and elderflowers, whereas from the group of flavonoids, different quercetin glycosides, kaempferol glycosides, isorhamnetin glycosides, and epicatechin were mainly quantified [27,42]. The anti-aging, anti-inflammatory, antiproliferative, anticancer, antibacterial, and antioxidant properties of phenolic compounds were reported by Albuquerque et al. [43] and Rahman et al. [44]. Among these compounds, ferulic acid was widely applied, mainly in skin care formulations, as a delayer of skin photoaging processes [45]. Additionally, its effect was verified against acute and chronic pathologies, e.g., intestinal ischemia, cancer, cardiovascular and skin diseases, diabetes, cochlear oxidative damage due to repeated noise exposure, and oxidative cellular stress in human dermal fibroblasts, as well as against neurodegenerative pathologies, especially Alzheimer’s disease [46]. Importantly, Rondini et al. [47] discovered that consuming ferulic acid through food had a greater impact on the human body than supplementing it with pure ferulic acid. In turn, quercetin is one of the most well-known flavonoids, which may reduce cellular aging by improving cell proliferation and the repair of the heterochromatin structure [48]. It is rapidly metabolized and excreted without accumulating in the body. Furthermore, it easily crosses the blood-brain barrier and exhibits neuroprotective activity, as well as playing a crucial role as an anti-inflammatory molecule [49].

Analyzing the antioxidant properties, it was found that the antioxidant capacity (AC) determined by the 2,2-diphenyl-1-picrylhydrazyl (DPPH) assay was higher in EF (10.74 μM TE/g and 11.31 μM TE/g for free and bound phenolic fractions, respectively) than in EB (9.19 μM TE/g and 8.38 μM TE/g, respectively) (Table 1). These results were consistent with TPC results. Unexpectedly, there was no correlation between the AC analyzed by the two analytical assays. The AC determined by the 2,2’-azino-bis(3-ethylbenzothiazoline-6-sulfonic acid) (ABTS) assay in EF was 72.33 μM TE/g in the free phenolic fraction and 58.07 μM TE/g in the bound phenolic fraction, while in EB the AC results were 187.73 μM TE/g and 10.53 μM TE/g, respectively.

On the contrary, a water extract from elderberries had significantly lower radical scavenging properties of DPPH^•^ (1911.6 μM TE/g) and ABTS^•+^ (2337.1 μM TE/g) than elderflower extract (DPPH = 4756.0 μM TE/g and ABTS = 6258.7 μM TE/g) [40]. Moreover, the ABTS values (3.20–39.59 μM TE/g) of various elderberry species and hybrids were considerably lower compared to the ABTS results (44.87–118.26 μM TE/g) of elderberry flowers [41].

However, the ABTS results for free and bound phenolic fractions of EF and EB were higher than the DPPH values. This suggests that the ABTS^•+^ radical cation is reactive towards most antioxidants (mainly free phenolics), including both hydrophilic and lipophilic compounds, whereas the DPPH^•^ radical can only be dissolved in organic media, especially in alcoholic media, which is an important limitation for the determination of hydrophilic antioxidants.

These findings are in line with the results of earlier studies, in which the antioxidant properties of elderflower and elderberry extracts are linked to the content of phenolic compounds [40,41,42].

### 2.2. Effect of Elderflower Dry Extract and Elderberry Juice Concentrate Additions on the Phenolic Composition of Gingerbread Cookies

The results of the chemical analysis of the gingerbread cookies showed that the addition of commercially available elder products had a generally positive effect on the content of phenolic compounds; however, the combined use of flower and juice products in the formulation was proven to be more favorable (Table 2).

There were clear differences in TPC between studied gingerbread cookies covered in chocolate: without additives (GC), enriched with elderflower dry extract (GCEF), and enriched with elderflower dry extract and elderberry juice concentrate (GCEFEB). The highest TPC result in the free phenolic fraction was determined in GCEF (277.44 μg/g), while the lowest was in GC (236.34 μg/g). The lower results of TPC in bound phenolic fraction than in free phenolic fraction were noted: 67.68 μg/g in GC, 67.77 μg/g in GCEF, and 68.79 μg/g in GCEFEB, respectively.

The addition of elderberry and elderflower extracts to dark chocolate also caused a plant extract type-dependent statistically significant increase in TPC results of fortified chocolate samples (11.7, 12.8, and 17.9 mg GA/g for chocolate without and with elderberry and elderflower extracts, respectively) [40]. Moreover, the enrichment of short crust cookies with elderflower, and wheat flour cookies with freeze-dried elderberries, increased the TPC from 1.01 mg GA/g and 91.26 mg/100 g in the control samples to 2.22 mg GA/g and 144.69 mg/100 g in the supplemented cookies [50,51]. Przybylski et al. [22] also found a high content of total polyphenols in gingerbread cakes after supplementation with tomato, beetroot, and pumpkin purée (39.02, 33.88, and 29.85 mg GA/100 g, respectively). On the contrary, similar TPC results were observed in gingerbread without (215.59 mg GA/100 g) and fortified with 3% chicken eggshell powder (214.01 mg GA/100 g) [3].

Six phenolic acids were identified in the studied phenolic fractions from gingerbread cookies, and ferulic acid was the main phenolic acid in all free phenolic fractions, with 54% in the GC, and GCEF, and 52% in the GCEFEB (Table 2). Ferulic acid was also the main phenolic acid in bound phenolic fractions in the GC (29%) and GCEF (28%), while *p*-coumaric acid was the most abundant in the GCEFEB (29%). Total phenolic acid contents ranged from 13.24 μg/g in the bound phenolic fraction of the GC to 18.40 μg/g in the free phenolic fraction of the GCEF. Furthermore, fourteen flavonoids were identified in phenolic fractions of the studied gingerbread cookies (Table 2). The highest content of flavonoids was observed in the bound phenolic fraction of the GCEFEB (27.39 μg/g), whereas the lowest flavonoid content was observed in the free phenolic fraction of the GC (9.63 μg/g). Isorhamnetin-3-*O*-glucoside was the main flavonoid in all free phenolic fractions, respectively, 55% in GC, 37% in GCEF, and 52% in GCEFEB, whereas epicatechin was the most abundant in the bound phenolic fraction, respectively, 85% in GC, 89% in GCEF, and 90% in GCEFEB.

When the effect of the elder products on the content of phenolic compounds in gingerbread cookies was examined, it was discovered that EF and EB increased the amounts of bound phenolic acids and both forms of flavonoids. In turn, the content of free phenolic acids was similar in the GCEF or lower in the GCEFEB compared to conventional gingerbread cookies (GC). This is probably a result of their binding by carbohydrates and proteins, and especially, ferulic acid and hydroxybenzoic acids seem to be more sensitive to heat treatment, which is consistent with the results of Liazid et al. [52].

The significant increase in the content of bound *p*-coumaric (up to 97%) and *p*-hydroxybenzoic acids (up to 55%) was noted after the addition of EF and EB (Table 2). In the case of flavonoids, these additives increased the content of free quercetin-3-*O*-glucoside (by 149% for EF and by 212% for EF + EB) and bound forms of epicatechin (by 26% and 11%, respectively) and apigenin (by 62% and 71%, respectively).

### 2.3. Effect of Elderflower Dry Extract and Elderberry Juice Concentrate Additions on the Antioxidant Properties of Gingerbread Cookies

**Figure 1:**
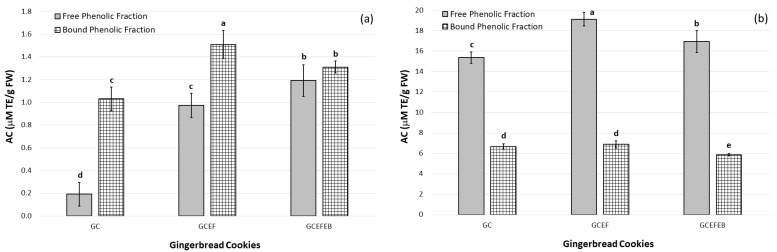
Antioxidant capacity (AC) results ($\bar{x\;}$± *SD*, mean value ± standard deviation) of gingerbread cookies covered in chocolate without additives (GC), and with elderflower dry extract (GCEF), as well as enriched with elderberry juice concentrate (GCEFEB), determined by DPPH (**a**) and ABTS (**b**) assays. Different letters (a–e), separately for each assay, indicate significant differences (one–way ANOVA and Tukey’s test, *p* ≤ 0.05).

Two radical scavenging assays (DPPH and ABTS) were used to receive reliable data on the effect of the addition of elderflower dry extract (EF) and elderberry juice concentrate (EB) to gingerbread cookies, and how this impacted their antioxidant properties (Figure 1). 

There were significant differences in the AC results of the same phenolic fraction determined by two different analytical assays. In the DPPH assay, the AC in the bound phenolic fraction was higher than that in the free phenolic fraction (1.03–1.51 μM TE/g vs. 0.19–1.19 μM TE/g, respectively), while the opposite was true in the ABTS assay (5.88–6.89 μM TE/g vs. 15.37–19.12 μM TE/g, respectively). It is noteworthy that the ABTS results were more than 14 times higher than those obtained by DPPH for the free phenolic fraction and 4 times higher for the bound phenolic fraction. 

Similarly, the ABTS values (433.9–1211.0 μM TE/g) of dark chocolates without and with elderberry and elderflower extracts were significantly higher than those of the DPPH (144.2–364.3 μM TE/g) [40]. This variability between the DPPH and ABTS results may be due to different affinities of the applied analytical methods toward hydrophobic and hydrophilic antioxidants. The ABTS assay is applicable for both lipophilic and hydrophilic antioxidants, while the hydrophobic nature of the DPPH^•^ radical limits the determination of hydrophilic antioxidants using the DPPH method. Moreover, ABTS^•+^ radical cations are more reactive than DPPH^•^ radicals due to the reactions of potential antioxidants with ABTS^•+^ involving both hydrogen atom transfer (HAT) and single electron transfer (SET), unlike the reactions with DPPH^•^ radicals, which mainly involve the HAT mechanism [40]. On the other hand, the structures of aromatic compounds provide a chromophoric system, which leads to interference in DPPH^•^ radicals. Therefore, the differences in antioxidant properties may be related to the chemical structure and type of antioxidants detected in gingerbread cookies.

The AC results confirmed that the bound phenolic fractions of gingerbread cookies without and with EF and EB were identified as more potent DPPH^•^ radical scavengers. In contrast, their free phenolic fractions more effectively scavenged the ABTS^•+^ radical cation (Figure 1). The AC increase in enriched gingerbread cookies can be explained by the fact that added plant extracts were a good source of bioactive phenolic compounds, including flavonoids (Table 1). Moreover, heat treatment during baking gingerbread cookies can enhance their antioxidant properties due to the formation of Maillard reaction products.

Previous research also reported that the DPPH and ABTS of the short crust cookies supplemented with elderflower significantly increased from 0.47 mg TE/g and 1.25 mg TE/g in the control sample to 2.10 mg TE/g and 3.45 mg TE/g in the enriched sample, respectively [50]. In addition, the DPPH (9.25 μM/g) and ABTS values (9.42 μM/g) of shortbread and wheat flour cookies fortified with elderberries were higher than scavenging activity of control samples (DPPH = 2.60 μM/g and ABTS = 1.11 μM/g) [51,53]. Unexpectedly, the addition of 3% eggshell powder to the gingerbread samples caused a decrease in their antioxidant properties (DPPH = 388.13 and 370.44 mg TE/100 g, and ABTS = 453.79 and 448.82 mg TE/100 g for control and enriched samples, respectively) [3].

The differences between the AC of free and bound phenolic fractions in the three types of gingerbread cookies were stable in the ABTS assay but unstable in the DPPH assay (Figure 1). It was found that the GCEFEB had the lowest differences between the AC of free and bound phenolic fractions in the DPPH assay (Figure 1a). Furthermore, the GCEF was characterized by the highest AC of free and bound phenolic fractions measured by the ABTS assay (Figure 1b), in contrast to the results obtained by the DPPH assay, where the GCEF had a lower AC of free phenolic fraction than the GCEFEB (Figure 1a).

### 2.4. Relationships between Phenolic Composition and Antioxidant Properties of Gingerbread Cookies

It can be noted that there were moderate relationships (r = 0.44–0.82) between the phenolic compound content and antioxidant properties of gingerbread cookies (Table 3).

The highest correlation coefficient (r = 0.82) was between the DPPH assay and total flavonoids, while the lowest correlation coefficient (r = 0.44) was calculated between the ABTS assay and total phenolic acids. Moreover, similar correlations were found between the ABTS values and total flavonoids (r = 0.68), DPPH results, and the total content of phenolic acids (r = 0.66). This data suggests that the content of polyphenols can be used as an indicator of the strength of antioxidant activity.

For comparison, higher correlation coefficients (r = 0.9993 and 0.9899) were calculated for relationships between radical scavenging activities determined by the DPPH and ABTS assays and TPC in short crust cookies supplemented with edible flowers [50]. Moreover, the correlations exhibited that the epicatechin and epigallocatechin contents in these cookies showed strong positive correlations with the DPPH (r = 0.8547 and 0.8762) and ABTS (r = 0.8740 and 0.8903) scavenging activities, even if their amount in the cookies was not very high.

### 2.5. Effect of Elderflower Dry Extract and Elderberry Juice Concentrate Additions on Advanced Glycation End Products’ Formation in Gingerbread Cookies

**Figure 2:**
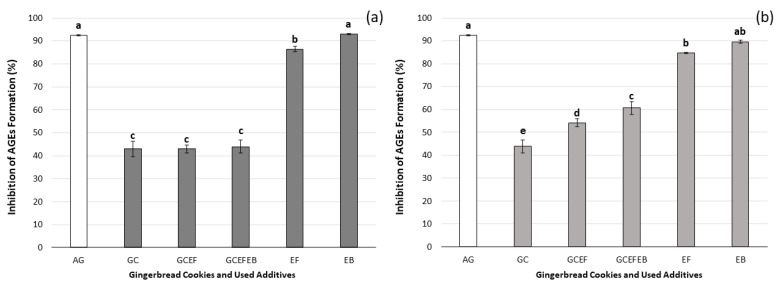
The inhibitory effects of gingerbread cookies covered in chocolate without additives (GC) and with elderflower dry extract (GCEF), as well as enriched with elderberry juice concentrate (GCEFEB); elderflower dry extract (EF); elderberry juice concentrate (EB); aminoguanidine solution (AG, as a positive control; 1 mM/L) against AGEs’ formation determined for free (**a**) and bound (**b**) phenolic fractions. Different letters (a–e), separately for each phenolic fraction, indicate significant differences (one-way ANOVA and Tukey’s test, *p* ≤ 0.05).

Dietary advanced glycation end products (AGEs) are formed in thermally treated foods as a result of the Maillard reaction. These products are essential to the total pool of AGEs produced in the living organism [54]. The inhibitory effects of the elder products and gingerbread cookies evaluated by the BSA-glucose model are shown in Figure 2. 

It was found that the inhibitory activity of the EB samples (free and bound fractions) revealed a higher value of AGEs’ inhibition (93.0% and 89.6%, respectively), while the inhibitory effect of aminoguanidine solution (AG) was 92.4%. The enrichment of gingerbread cookies with dry elderflower extract (GCEF) and elderberry juice concentrate (GCEFEB) did not significantly increase the inhibitory activity against AGEs’ formation in free phenolic fractions compared to the control cookies. However, bound phenolic fraction samples were characterized by higher inhibitory activity values, and the highest value was observed in the bound fraction obtained from cookies enriched with EF and EB (Figure 2). This phenomenon may be related to hydrolysis, which resulted in the release of more compounds with an inhibitory activity against AGEs. The increased contents of epicatechin and/or naringenin present were observed in the bound fraction. Previously published studies also showed that epicatechin and naringenin, as major dietary flavonoids, could inhibit the formation of AGEs [55,56]. In addition, other phenolic compounds derived from plant extracts also inhibited the formation of AGEs. Their main mechanism is inhibiting the production of free radicals in the glycation process [57,58].

The correlation studies demonstrated that the inhibitory effects of analyzed samples against the formation of AGEs were correlated with their bioactive compound contents (for free fractions: TPC/AGEs inhibition, r = 0.77; the sum of phenolic acid/AGEs inhibition, r = 0.83; the sum of flavonoids/AGEs inhibition, r = 0.94; and for bound fractions: TPC/AGEs inhibition, r = 0.53; the sum of phenolic acid/AGEs inhibition, r = 0.55; the sum of flavonoids/AGEs inhibition, r = 0.72) and AC (ABTS assay/AGEs inhibition, r = 0.60; DPPH assay/AGEs inhibition, r = 0.95) determined in the samples. Our data on the anti-glycation effect are in agreement with the previously published research [54,59].

### 2.6. Effect of Elderflower Dry Extract and Elderberry Juice Concentrate Additions on the Physical Characteristics and Sensory Acceptance of Gingerbread Cookies

Physical measurements indicated that the addition of EF and EB did not affect the height and diameter of the gingerbread cookies (Table 4). Moreover, as was to be expected, the addition of EB caused an increase in the weight of the gingerbread cookies.

Similarly, the addition of elderberry and other fruit pomaces had a negligible effect on the geometric features of shortbread cookies. All baked cookies preserved their round shape and diameter (57.22 and 56.75–57.54 mm for cookies without and with fruit pomace, respectively) [53].

On the other hand, the presence of EF and EB additives in the GCs had no significant impact on surface color, defined using lightness (L*) and chromaticity parameters, redness (a*) and yellowness (b*) (Table 4). However, the incorporation of gingerbread cookies with EF and EB significantly affected the cross-section color by decreasing L* value and increasing a* and b* values (except GCEFEB). Therefore, the lightness of the GCEFEB was reduced from 50.50 and 75.82 to 49.29 and 68.24 for the surface and cross-section color, respectively (Table 4). Moreover, the a* (−1.10 and −1.40) and b* (27.89 and 50.20) values for the surface and cross-section of the GCEF were higher compared with the control GC (a* = −1.26 and −1.96, b* = 26.94 and 48.46, respectively). On the contrary, a decrease in the cross-section values of a* (−2.62) and b* (42.94) was observed after the incorporation of EF and EB (Table 4).

In a previous article, the lightness (L* = 57.22) and redness (a* = −2.39) of cookies with elderberry pomace were also lower than the L* (94.82) and a* (−1.76) values for the control cookies [53]. However, the yellowness (b*) of these enriched cookies increased from 18.32 to 22.19. Nevertheless, all color parameters L*, a* and b* decreased with the addition of freeze-dried elderberry to gluten-free wafer batter and wafer sheets [60]. Although elderberry flower powder contained color pigments, the low differences in the color parameters of the fortified gingerbread cookies were affected by their dark color compared with the shortbread cookies [53] and wafers [60].

It is well known that color parameters clearly affect consumers’ acceptance of the visual appearance of final food products. As can be seen in Table 5, there were differences in the sensory characteristics between each type of the studied gingerbread cookies, resulting from the raw materials used.

It is noteworthy that for such attributes as flavor, and texture, significant differences between the GCEFEB, GCEF, and GC were demonstrated. The highest results for these attributes were obtained for the GCEFEB: 7.71 for flavor and 7.10 for texture on a 9-point scale. A slightly lower rating for flavor was noted in the GCEF (6.98), whereas a very low score was obtained for the GC (2.88). The texture attributes for the GCEF and GC were also significantly lower than the GCEFEB and were 5.10 and 3.38, respectively. In opposition to flavor and texture attributes, the color and odor scores obtained for the GCEFEB, GCEF, and GC were comparable, and they were assessed, respectively, at 7.22, 7.24, and 7.38 for the color attribute and 8.39, 8.38, and 7.64 for the odor attribute. Regardless of the above ratings, the overall acceptability was assessed for each type of gingerbread cookie (Table 5). The highest score (7.97), which corresponded to the classification “liked very much”, was obtained by the GCEFEB. A slightly lower score (7.16) was had for the GCEF, and a significantly lower score (3.17), which corresponded to the classification “disliked moderately”, was found for the GC.

The evaluators paid particular attention to the wide range of flavor characteristics, which varied depending on the type of gingerbread cookie. Gingerbread cookies covered in chocolate (GC) scored the lowest in terms of texture and flavor, at a level of approximately “disliked moderately”. These properties in the enriched gingerbread cookies were more acceptable, and they were described as “liked moderately” for the GCEFEB, while they scored as “neither liked nor disliked” (texture) or “liked slightly” (flavor) for the GCEF.

Other studies also showed that supplementing gingerbread with plant powders positively affected its sensory characteristics. For example, Ghendov-Mosanu et al. [19] introduced 2% and 4% of rose hip pulp powder to gingerbread and observed that these additives improved its general characteristics, but a lower concentration was preferred due to the specific smell and taste of rose hips. In addition, Sanokulovich et al. [20] established that the addition of powders from medicinal plants in dosages ranging from 0.5% to 2.0% was more recommended for gingerbread. They found that increasing the concentration of peppermint and camel thorn powders led to the appearance of bitterness in the taste, darkening of the crumb, and a deterioration of its porosity. Furthermore, the sensory evaluation of gingerbread fortified with 3% eggshell powder [3] also exhibited moderate–high (7.00–7.80 on a 9-point structured hedonic scale) consumer acceptability. There were no significant differences between the sensory profiling (appearance, aroma, texture, and taste) of gingerbread with 3% eggshell powder and the control sample. Consequently, the addition of pumpkin, tomato, and beetroot to gingerbread at a level of up to 25% provided an adequate effect on the sensory quality of the enriched products while still being acceptable to consumers [22]. The tested attributes in terms of appearance liking, aroma liking, texture liking, taste/flavor liking, and the overall liking of gingerbreads enriched with tomato and pumpkin were rated highly (above a 6.00 score). In turn, Tańska et al. [53] showed that the addition of elderberry pomace to shortbread cookies caused a decrease in sensory acceptance. The cookies were characterized by a more perceptible taste and aroma and were sourer.

On the contrary, our results of the hedonic evaluation of the GC, GCEF, and GCEFEB by untrained panelists demonstrated that the GCEF and GCEFEB stood out in sensory acceptance. The GCEFEB and GCEF were pleasant and well-accepted by panelists. Unexpectedly, the enrichment of the GC with EF and EB rich in free and bound phenolic compounds (Table 2) improved the sensory quality, making the new products more accepted. Phenolic compounds are closely associated with sensory quality. Oxidative changes during processing can decrease the sensory evaluation of the final products and reduce the willingness to purchase them [61]. However, this study confirmed that the production of GCs enriched with EF and EB, having high nutritional and antioxidant properties and good sensory acceptance, can be an appropriate strategy from a commercial point of view.

**Figure 3:**
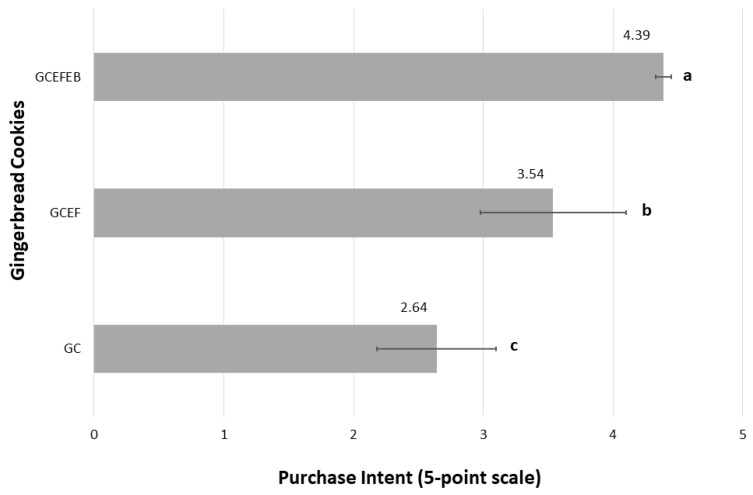
Average ratings of purchase intent of gingerbread cookies covered in chocolate without additives (GC), and with elderflower dry extract (GCEF), as well as enriched with elderberry juice concentrate (GCEFEB). Purchase intent was based on the 5-point scale (1 = certainly would not buy, 2 = probably would not buy, 3 = might or might not buy, 4 = probably would buy, and 5 = certainly would buy). Different letters (a–c) indicate significant differences (one-way ANOVA and Tukey’s test, *p* ≤ 0.05).

The desire to buy locally produced gingerbread cookies was directly proportional to sensory assessment. In the context of the proposed products’ purchase intent frequency, the GCEFEB received the highest scores (4.39 ± 0.06 on a 5-point scale) and was qualified as “certainly would buy” (Figure 3).

As can be seen, both EF and EB had a positive effect on the purchase intent of the new gingerbread cookies. It is noteworthy that the results of overall acceptability (Table 5) were correlated with purchase intent, which is very valuable information from a commercial point of view. The GCEFEB received 4.39 points, which is a result indicating great potential for this product on the market. In the same study, the GCEF had moderate purchase intent scores of 3.54 points, being largely qualified with “probably would buy”. The lowest purchase intention (2.64 points) was declared for the GC, which suggests that the product in this form should not be launched on the market. This phenomenon is probably related to the unusual aroma of cakes with elderberry products, which may be attractive to consumers. In addition, elderberries have a low sugar content compared to other fruit species [41]. Therefore, they are an ideal additive that does not increase the caloric content of a new product.

## 3. Materials and Methods

### 3.1. Chemicals

Standard phenolic compounds (purity > 97%), including phenolic acids (benzoic, caffeic, ferulic, *p*-coumaric, *m*-hydroxybenzoic, *p*-hydroxybenzoic) and flavonoids (apigenin, D-catechin, epicatechin, kaempferol, naringenin, quercetin), Trolox (6-hydroxy-2,5,7,8-tetramethylchromane-2-carboxylic acid), DPPH (2,2-diphenyl-1-picrylhydrazyl), ABTS (2,2′-azino-bis(3-ethylbenzothiazoline-6-sulphonic acid)), and HPLC-grade solvents and reagents such as acetonitrile, ammonium formate, bovine serum, formic acid, and glucose were purchased from Sigma Aldrich (Poznań, Poland). Analytical-grade reagents such as acetic acid, Folin–Ciocalteau (F-C) reagent, methanol, phosphate buffer, potassium dithionite, sodium carbonate, and sodium hydroxide were supplied by Chempur (Piekary Śląskie, Poland). 

### 3.2. Materials

Three types of gingerbread cookies were produced through a traditional technological process in the Confectionery Factory (Fabryka Cukiernicza Kopernik S.A., Toruń, Poland). Ingredients and photos of the samples are presented in Table 6.

Samples of gingerbread cookies (GC) were supplemented with powdered elderberry flower extract (EF) supplied by Greenvit Botanical Extracts Manufacturer in Zambrów (Poland), and concentrated elderberry fruit juice (EB) supplied by DÖHLER Natural Food & Beverage Ingredients (Darmstadt, Germany). The GCs covered in chocolate but without elder products were used as a control sample. The materials were kept in a polyethylene (PE) bag in a cool and dry place until the research was performed.

### 3.3. Extraction of Free and Bound Phenolic Fractions 

Free and bound phenolic compounds were extracted using the method by Šťastná et al. [50] with some modifications. The ground gingerbread samples (laboratory mill type A 10; IKA Labortechnik, Staufen, Germany) were weighed (10 ± 0.001 g) into dark flasks, and 40 mL of 80% methanol was added to each flask. In turn, the samples of EF and EB were lower in weight (2 ± 0.001 g), and 20 mL of 80% methanol was added into each flask. The mixtures were sonicated for 1 h in an ultrasonic bath (InterSonic, Olsztyn, Poland) and then they were centrifuged at 13,000× *g* for 15 min (type 5810R; Eppendorf AG centrifuge, Hamburg, Germany). Supernatants were used as free phenolic fractions.

The precipitates after free phenolic extraction were used for the extraction of bound phenolics. Briefly, 25 mL of 0.1 M NaOH was added to each precipitate and left in an ultrasonic bath (InterSonic) for 1 h. The mixtures were centrifuged at 13,000× *g* for 15 min (type 5810R; Eppendorf AG centrifuge). Then, the pH of the supernatants was adjusted to the range of pH 3–5 using 6 M HCl and centrifuged again at 13,000× *g* for 15 min (type 5810R; Eppendorf AG centrifuge). These supernatants were used as bound phenolic fractions. 

### 3.4. Determination of Total Phenolic Content

The total content of phenolic compounds (TPC) was determined spectrophotometrically with the Folin–Ciocalteau reagent, according to Zakrzewski et al. [62], with some modifications. The color reaction was carried out by adding the Folin–Ciocalteau reagent (0.25 mL), 14% sodium carbonate (1.5 mL), and distilled water (3.15 mL) to the polyphenol extract (0.1 mL). After mixing, the solution was left for 60 min, and absorbance was measured against the reagent sample (without the phenolic extract) at a wavelength of 720 nm using a FLUOstar Omega microplate reader (BMG LABTECH, Offenburg, Germany). The TPC analysis was performed in triplicate for each sample, and the results were expressed as the μg catechin equivalent (CE) per 1 g of sample fresh weight (FW). The following equation was used for calculating the TPC: Absorbance at 720 nm = 2.9023x + 0.0521 (R² = 0.9986).

### 3.5. HPLC Determination of Phenolic Compound Content

The analysis of individual phenolic compounds was performed according to the methodology described by Zakrzewski et al. [62] with some modifications. The qualitative and quantitative analyses of the polyphenols were carried out using an ultra-high performance liquid chromatography (UHPLC) system (Nexera XR, Shimadzu, Japan) coupled with a diode area detector (DAD) and mass spectrometer (LCMS-2020, Shimadzu, Japan). The measurement parameters were as follows: 0.01% formic acid in water with 1 mM ammonium formate (eluent A) and 0.01% formic acid in 95% acetonitrile solution with 1 mM ammonium formate (eluent B); flow rate 0.37 mL/min; scanning in negative ionization; column Kinetex (2.6 μm particle size; 100 mm × 4.6 mm) (Phenomenex, Torrance, CA, USA); oven temperature was 40 °C; sample injection volume 10 µL. An analysis was conducted in the selected ion monitoring mode (SIM). Analyzed compounds were identified according to their qualitative ions, retention times, and λ_max_, as summarized in Table S1. The quantities of polyphenols were calculated from the UHPLC-DAD-MS peak area against commercially available standards (*p*-coumaric, *m*-hydroxybenzoic, *p*-hydroxybenzoic, caffeic, ferulic and benzoic acids, epicatechin, quercetin, apigenin, and naringenin), while the glycosidic forms of quercetin, kaempferol, myricetin, and isorhamnetin were expressed as quercetin or kaempferol equivalents.

The least squares method was used to obtain the equations of the calibration curves (y = ax + b). A goodness of fit was given by the coefficient of determination (R^2^), which is evidence of linearity for all analyzed phenolic compounds in the concentration range from 0.01 to 150 μg/mL (Table S1). The limit of detection (LOD) and limit of quantification (LOQ) values were calculated based on the signal-to-noise (S/N) ratio. The level of noise was measured from the chromatograms obtained for the standard solutions at the lowest concentration level. The LOD was calculated as being three times higher than the level of noise, and the LOQ was equal to ten times the noise level. The phenolic compounds were determined in triplicate for each sample and expressed as μg per 1 g of sample FW.

### 3.6. Determination of Antioxidant Properties 

The antioxidant capacity (AC) of elder products and gingerbread cookies was studied using 2,2-diphenyl-1-picrylhydrazyl (DPPH) and 2,2’-azino-bis(3-ethylbenzothiazoline-6-sulfonic acid) (ABTS) assays, according to Horszwald and Andlauer [63] with some modifications. Both antioxidant tests were performed in triplicate for each sample.

In the case of the DPPH assay, each extract (50 μL) was added to a DPPH solution (450 μL, 0.2 mmol/L in methanol), and the mixture was shaken and incubated in the dark at room temperature for 30 min. Absorbance was measured at 517 nm against methanol using a FLUOstar Omega microplate reader. The AC was determined based on a curve of the % DPPH radical scavenging activity of different Trolox concentrations (within the range of 0.05 to 2 mmol/L) in methanol and expressed as μM TE (Trolox equivalent) per 1 g of sample FW. The following calibration equation was used: %DPPH = 242.66x + 7.9544 (R² = 0.9973).

According to the ABTS assay, the 7 mmol/L aqueous solution of ABTS (10 mL) and the 51.4 mmol/L aqueous solution of potassium dithionite (K_2_S_2_O_4_) (0.5 mL) were mixed in order to obtain an ABTS^•+^ radical cation solution with an absorbance value of 0.7 at λ = 734 nm. Then, 20 μL of each extract was added to 1480 μL of ABTS^•+^ solution. The reaction was performed at 30 °C in the dark for 6 min. After this time, the values of absorbance were recorded using a microplate reader (FLUOstar Omega). The Trolox solution (stock solution, 1 mmol/L) was used for calibration, and the AC was expressed as the μM TE per 1 g of sample FW. The following calibration equation was used: %ABTS = 104.03x + 1.1905 (R² = 0.9999).

### 3.7. Anti-Glycation Assay

To determine the anti-glycation properties of elder products and gingerbread cookies, the bovine serum (BSA)-glucose model was used to describe the AGEs’ formation. In the first step, the obtained extracts were dried under nitrogen. After drying, samples were dissolved in this same amount of phosphate buffer (0.1 M, pH 7.4) and used directly for the anti-glycation assay as described by Przygodzka and Zieliński [54]. Fluorescence intensity (excitation wave 330 nm and emission wave 410 nm) was measured using a microplate reader (FLUOstar Omega). The percent inhibition of AGEs’ formation by a sample, or the aminoguanidine (AG) solution (1 mM) used as a positive control, was calculated. The analysis was performed in triplicate for each sample.

### 3.8. Measurements of Physical Parameters

The gingerbread cookies were characterized by weight, size (diameter and height) and color parameters. The measurements were performed 2 h after baking for 10 cookies in each sample.

The weight of the cookies was determined with an electronic weighing balance (type 125A, Precisa Gravimetrics AG, Dietikon, Switzerland). The height and diameter of the cookies were measured using a vernier caliper.

The color was determined on the cookie’s surface and its cross-section. A digital image analysis (DIA) was used for these measurements. The equipment consisted of a charge-coupled device (CCD) color camera (DXM-1200, Nikon Instruments, Melville, NY, USA), a Kaiser RB 5004 HF–High Frequency Daylight Copy Light set with 4 × 36 W fluorescent light tubes (color temperature about 5400 K) (Kaiser Fototechnik GmbH and Co., KG, Buchen, Germany), and Laboratory Universal Computer Image Analysis (LUCIA) G v. 4.8 software (Laboratory Imaging, Prague, Czech Republic). The results were expressed in the CIEL*a*b* color model, where the L* parameter represented lightness (in the range of 0–100, from the darkest black to the brightest white, respectively), the a* parameter represented green/red color (negative/positive values), and the b* parameter represented blue/yellow color (negative/positive values) [53].

### 3.9. Sensory Acceptance Test

Sensory analysis of the gingerbread cookies was performed using an effective acceptance test with 112 untrained panelists (54 males and 58 females) in the age range of 18–63 recruited among customers and employees of the Confectionery Factory (Fabryka Cukiernicza Kopernik S.A., Toruń, Poland). The sensory test was conducted two days after the baking trials using a 9-point hedonic scale (1 = disliked extremely, 2 = disliked very much, 3 = disliked moderately, 4 = disliked slightly, 5 = neither liked nor disliked, 6 = liked slightly, 7 = liked moderately, 8 = liked very much, and 9 = liked extremely), according to Wichchukit and O’Mahony [64]. The participants were asked to assess the following attributes: liking of color, liking of odor, liking of texture, liking of flavor, and overall acceptability. Additionally, the purchase intent was evaluated using a 5-point scale (1 = certainly would not buy, 2 = probably would not buy, 3 = might or might not buy, 4 = probably would buy, and 5 = certainly would buy). For this reason, there was a question: “How likely is it that you will buy this product if it will be available in stores?” at the end of the questionnaire card. Each untrained panelist evaluated a total of three types of gingerbread cookies in an odor-free plastic container with a lid labeled with a 3-digit code in a randomized order to avoid an order effect [65]. The panelists used warm dark tea to rinse their mouths between samples testing.

### 3.10. Data Analysis

All obtained results were analyzed using Statistica 13.0 PL software (StatSoft, Kraków, Poland) at a significance level of *p* ≤ 0.05. They were checked for normal distribution (Shapiro–Wilk’s test) and homogeneity of variances (Levene’s test). The differences between samples were determined using a one-way ANOVA with a Tukey’s test. Additionally, Pearson’s correlation coefficients (r) were calculated to determine the relationships between the antioxidant properties and the contents of phenolic compounds.

## 4. Conclusions

The present study has generated important information related to the increase in phenolic compounds as well as the in vitro antioxidant properties of gingerbread cookies (GC) by introducing elderflower dry extract (EF) and elderberry juice concentrate (EB) into the formulation. Gingerbread cookies with the addition of both elder products were characterized by the highest content of flavonoids. Regardless of the enrichment applied, the bound flavonoids were dominant. In contrast, these additives did not increase the content of free phenolic acids. Furthermore, the content of bound phenolic acids was higher in the enriched GC, with a slightly greater concentration when only EF was incorporated into the chocolate coating. The higher content of flavonoids was strongly correlated with the AC. The anti-glycation properties of the elder products were confirmed for the bound phenolic fraction, which is probably a result of the higher content of flavonoids in this phenolic fraction, especially epicatechin and naringenin. In addition, the positive influence of complex elder products on the formation of the sensory properties of the gingerbread cookies was revealed. It was noted that such attributes as flavor and texture were significantly improved by these additives.

It can be concluded that it is possible to produce gingerbread that maintains good sensory qualities and exhibits the characteristics of a health-promoting functional food, due to its antioxidant and anti-glycation properties, thanks to the addition of polyphenols from elder products.

## Figures and Tables

**Table 1:** Content of free and bound phenolic compounds and their antioxidant capacity ($\bar{x\;}$ ± *SD*) for elderflower dry extract (EF) and elderberry juice concentrate (EB).

	EF		EB	
	Free Phenolic Fraction	Bound Phenolic Fraction	Free Phenolic Fraction	Bound Phenolic Fraction
TPC (μg CE/g FW)	831.14 ± 12.23 ^a^	850.94 ± 13.21 ^a^	437.69 ± 16.69 ^b^	90.58 ± 7.92 ^c^
Content of Phenolic Acids (μg/g FW)				
Benzoic Acid	26.84 ± 0.74 ^a^	20.26 ± 1.02 ^b^	24.14 ± 0.24 ^a^	<LOD
Caffeic Acid	<LOD	4.80 ± 0.29 ^a^	2.14 ± 0.06 ^b^	<LOD
*p*-Coumaric Acid	<LOD	3.43 ± 0.12 ^b^	<LOD	18.75 ± 0.58 ^a^
Ferulic Acid	10.27 ± 0.24 ^c^	442.79 ± 26.17 ^a^	148.53 ± 0.65 ^b^	11.03 ± 0.56 ^c^
*m*-Hydroxybenzoic Acid	2.65 ± 0.07 ^a^	1.86 ± 0.06 ^b^	<LOD	<LOD
*p*-Hydroxybenzoic Acid	15.08 ± 1.03 ^a^	11.34 ± 0.42 ^b^	2.08 ± 0.08 ^c^	<LOD
Sum of Phenolic Acids	54.84 ± 0.87 ^c^	484.50 ± 26.96 ^a^	176.89 ± 0.56 ^b^	39.78 ± 0.93 ^d^
Content of Flavonoids (μg/g FW)				
Apigenin	<LOD	0.62 ± 0.01 ^b^	<LOD	6.66 ± 0.41 ^a^
Epicatechin	<LOD	13.99 ± 0.49 ^b^	15.80 ± 1.29 ^b^	32.06 ± 1.78 ^a^
Isorhamnetin-3-*O*-glucoside	23.28 ± 0.70 ^a^	7.67 ± 0.72 ^b^	<LOD	<LOD
Isorhamnetin-3-*O*-rutinoside	1.62 ± 0.04 ^a^	<LOD	<LOD	<LOD
Kaempferol-3-*O*-rutinoside	35.29 ± 2.01 ^a^	14.23 ± 0.27 ^b^	11.67 ± 0.48 ^c^	<LOD
Myricetin-3-*O*-glucoside	4.57 ± 0.27	<LOD	<LOD	<LOD
Myricetin-3-*O*-rutionoside	2.27 ± 0.16 ^b^	3.64 ± 0.14 ^a^	<LOD	<LOD
Naringenin	4.40 ± 0.05 ^b^	4.26 ± 0.01 ^b^	<LOD	5.45 ± 0.08 ^a^
Quercetin	1.31 ± 0.01 ^b^	<LOD	22.71 ± 0.74 ^a^	<LOD
Quercetin-3-*O*-glucoside	131.17 ± 7.66 ^a^	60.41 ± 3.95 ^c^	98.02 ± 2.94 ^b^	0.95 ± 0.05 ^d^
Quercetin-3-*O*-vicianoside	<LOD	0.90 ± 0.02	<LOD	<LOD
Quercetin-*O*-hexosyl-*O*-hexoside	6.17 ± 0.07 ^a^	2.18 ± 0.14 ^b^	<LOD	<LOD
Quercetin-*O*-pentosyl-hexoside	<LOD	1.75 ± 0.05	<LOD	<LOD
Quercetin-dihexoside	4.41 ± 0.13	<LOD	<LOD	<LOD
Sum of Flavonoids	219.07 ± 9.52 ^a^	109.63 ± 4.36 ^c^	148.20 ± 3.78 ^b^	45.12 ± 2.04 ^d^
Antioxidant Capacity (μM TE/g FW)				
DPPH Assay	10.74 ± 0.33 ^b^	11.31 ± 1.05 ^b^	9.19 ± 0.38 ^a^	8.38 ± 0.86 ^c^
ABTS Assay	72.33 ± 1.74 ^b^	58.07 ± 1.87 ^c^	187.73 ± 4.77 ^a^	10.53 ± 0.74 ^d^

*n* = 3; $\bar{x\;}\pm SD$—mean value ± standard deviation; LOD—limit of detection; DPPH—2,2-diphenyl-1-picrylhydrazyl; ABTS—2,2’-azino-bis(3-ethylbenzothiazoline-6-sulfonic acid); FW—fresh weight; different letters (^a–d^) within the same line indicate significant differences (one-way ANOVA and Tukey’s test, *p* ≤ 0.05).

**Table 2:** Content of free and bound phenolic compounds and their antioxidant capacity ($\bar{x\;}$ ± *SD*) for gingerbread cookies covered in chocolate without additives (GC), and with elderflower dry extract (GCEF), as well as enriched with elderberry juice concentrate (GCEFEB).

Phenolic Compounds	GC		GCEF		GCEFEB	
	Free Phenolic Fraction	Bound Phenolic Fraction	Free Phenolic Fraction	Bound Phenolic Fraction	Free Phenolic Fraction	Bound Phenolic Fraction
TPC (μg CE/g FW)	236.34 ± 5.87 ^c^	67.68 ± 3.62 ^d^	277.44 ± 8.50 ^a^	67.77 ± 9.03 ^d^	256.16 ± 9.55 ^b^	68.79 ± 6.44 ^d^
Content of Phenolic Acids (μg/g FW)						
Benzoic Acid	6.41 ± 0.00 ^a^	<LOD	6.57 ± 0.13 ^a^	<LOD	6.01 ± 0.09 ^a^	<LOD
Caffeic Acid	<LOD	<LOD	<LOD	<LOD	<LOD	<LOD
*p*-Coumaric Acid	<LOD	2.27 ± 0.08 ^c^	<LOD	4.27 ± 0.10 ^b^	<LOD	4.48 ± 0.02 ^a^
Ferulic Acid	9.69 ± 0.76 ^a^	3.89 ± 0.16 ^d^	9.99 ± 0. 48 ^a^	4.45 ± 0.28 ^c^	7.62 ± 0.46 ^b^	3.63 ± 0.16 ^d^
*m*-Hydroxybenzoic Acid	0.64 ± 0.05 ^c^	1.98 ± 0.09 ^a^	0.58 ± 0.03 ^c^	1.41 ± 0.09 ^b^	<LOD	1.53 ± 0.09 ^b^
*p*-Hydroxybenzoic Acid	1.34 ± 0.07 ^b^	1.11 ± 0.09 ^c,d^	1.26 ± 0.02 ^c^	1.70 ± 0.12 ^a^	0.99 ± 0.03 ^d^	1.72 ± 0.07 ^a^
Sum of Phenolic Acids	18.08 ± 0.77 ^a^	13.24 ± 0.10 ^d^	18.40 ± 0.63 ^a^	15.82 ± 0.40 ^b^	14.62 ± 0.50 ^c^	15.36 ± 0.19 ^b^
Content of Flavonoids (μg/g FW)						
Apigenin	<LOD	0.79 ± 0.04 ^b^	<LOD	1.00 ± 0.06 ^a^	<LOD	0.88 ± 0.05 ^b^
Epicatechin	<LOD	14.43 ± 1.16 ^c^	4.23 ± 0.18 ^d^	23.33 ± 0.33 ^b^	<LOD	24.65 ± 0.13 ^a^
Isorhamnetin-3-*O*-glucoside	5.26 ± 0.16 ^b^	<LOD	6.04 ± 0.34 ^a^	<LOD	6.50 ± 0.07 ^a^	<LOD
Isorhamnetin-3-*O*-rutinoside	<LOD	<LOD	<LOD	<LOD	<LOD	<LOD
Kaempferol-3-*O*-rutinoside	<LOD	<LOD	<LOD	<LOD	<LOD	<LOD
Myricetin-3-*O*-glucoside	<LOD	<LOD	<LOD	<LOD	<LOD	<LOD
Myricetin-3-*O*-rutionoside	<LOD	<LOD	<LOD	<LOD	<LOD	<LOD
Naringenin	<LOD	1.81 ± 0.01 ^a^	<LOD	1.85 ± 0.06 ^a^	<LOD	1.85 ± 0.06 ^a^
Quercetin	<LOD	<LOD	<LOD	<LOD	<LOD	<LOD
Quercetin-3-*O*-glucoside	0.97 ± 0.05 ^c^	<LOD	2.42 ± 0.02 ^b^	<LOD	3.04 ± 0.06 ^a^	<LOD
Quercetin-3-*O*-vicianoside	1.87 ± 0.13 ^b^	<LOD	2.19 ± 0.12 ^a^	<LOD	1.78 ± 0.09 ^b^	<LOD
Quercetin-*O*-hexosyl-*O*-hexoside	<LOD	<LOD	<LOD	<LOD	<LOD	<LOD
Quercetin-*O*-pentosyl-hexoside	1.52 ± 0.07 ^a^	<LOD	1.26 ± 0.04 ^b^	<LOD	1.15 ± 0.05 ^c^	<LOD
Quercetin-dihexoside	<LOD	<LOD	<LOD	<LOD	<LOD	<LOD
Sum of Flavonoids	9.63 ± 0.38 ^e^	17.03 ± 1.19 ^c^	16.13 ± 0.36 ^c^	26.19 ± 0.35 ^b^	12.47 ± 0.09 ^d^	27.39 ± 0.11 ^a^

*n* = 3; $\bar{x\;}\pm SD$—mean value ± standard deviation; LOD—limit of detection; different letters (^a–e^) within the same line indicate significant differences (one-way ANOVA and Tukey’s test, *p* ≤ 0.05).

**Table 3:** Pearson’s correlation coefficients (r) between antioxidant properties and the content of phenolic compounds of studied gingerbread cookies.

Phenolic Compounds Content	Antioxidant Properties	
	DPPH Assay	ABTS Assay
Total Phenolic Compounds	0.77 *	0.57
Total Phenolic Acids	0.66 *	0.44
Total Flavonoids	0.82 *	0.68 *

*—statistically significant at *p* ≤ 0.05.

**Table 4:** Physical parameters ($\bar{x\;}$ ± *SD*) of gingerbread cookies covered in chocolate without additives (GC), and with elderflower dry extract (GCEF), as well as enriched with elderberry juice concentrate (GCEFEB).

Parameter	GC	GCEF	GCEFEB
Weight (g)	14.03 ± 0.34 ^b^	14.07 ± 0.59 ^b^	19.06 ± 0.86 ^a^
Height (mm)	23.06 ± 0.59 ^a^	23.00 ± 0.30 ^a^	22.36 ± 0.16 ^a^
Diameter (mm)	54.55 ± 0.82 ^a^	54.66 ± 0.75 ^a^	54.50 ± 0.56 ^a^
Surface Color			
L*	50.50 ± 0.61 ^a^	49.88 ± 0.54 ^a^	49.29 ± 0.78 ^a^
a*	−1.26 ± 0.23 ^a^	−1.10 ± 0.34 ^a^	−1.07 ± 0.37 ^b^
b*	26.94 ± 0.74 ^a^	27.89 ± 0.70 ^a^	27.58 ± 0.36 ^a^
Cross-section Color			
L*	75.82 ± 0.45 ^a^	72.48 ± 0.44 ^b^	68.24 ± 3.24 ^c^
a*	−1.96 ± 0.94 ^ab^	−1.40 ± 0.29 ^a^	−2.62 ± 0.34 ^b^
b*	48.46 ± 0.13 ^b^	50.20 ± 0.60 ^a^	42.94 ± 0.53 ^c^

*n* = 10; $\bar{x\;}\pm SD$—mean value ± standard deviation; different letters (^a–c^) within the same column indicate significant differences (one-way ANOVA and Tukey’s test, *p* ≤ 0.05).

**Table 5:** Mean sensory scores ($\bar{x\;}$ ± *SD*) for the color, odor, texture, flavor, and overall acceptability of gingerbread cookies covered in chocolate without additives (GC), and with elderflower dry extract (GCEF), as well as enriched with elderberry juice concentrate (GCEFEB).

Sample	Color	Odor	Texture	Flavor	Overall Acceptance
GC	7.38 ± 1.31 ^a^	7.64 ± 0.90 ^b^	3.38 ± 1.14 ^c^	2.88 ± 1.08 ^b^	3.17 ± 0.91 ^b^
GCEF	7.24 ± 0.94 ^a^	8.38 ± 0.62 ^a^	5.10 ± 0.80 ^b^	6.98 ± 0.69 ^a^	7.16 ± 0.67 ^a^
GCEFEB	7.22 ± 0.93 ^a^	8.39 ± 0.67 ^a^	7.10 ± 1.00 ^a^	7.71 ± 1.03 ^a^	7.97 ± 0.95 ^a^

*n* = 112; $\bar{x\;}\pm SD$—mean value ± standard deviation; color, odor, texture, flavor and overall acceptability are based on the 9-point hedonic rating scale system, with anchoring point, 1—“disliked extremely” and 9—“liked extremely”; different letters (^a–c^) within the same column indicate significant differences (one-way ANOVA and Tukey’s test, *p* ≤ 0.05).

**Table 6:** Photos and ingredients of the three types of gingerbread cookies produced.

	Gingerbread Covered in Chocolate	Gingerbread Covered in Chocolate Fortified by Elderflower Dry Extract	Gingerbread Covered in Chocolate Fortified by Elderflower Dry Extract and Filled with Elderberry Juice Concentrate
Code	GC	GCEF	GCEFEB
Photo	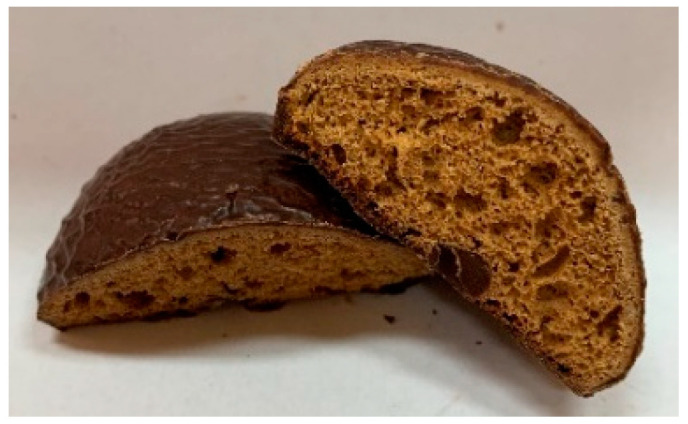	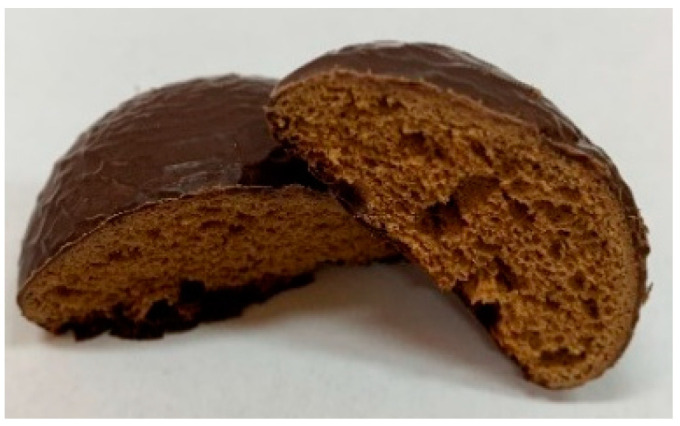	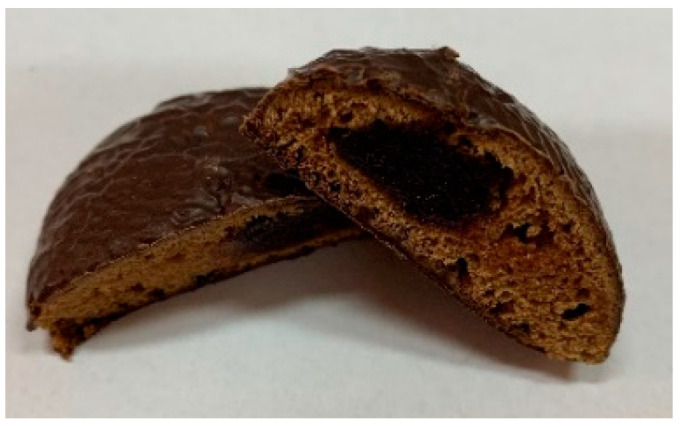
Ingredients	wheat flour, chocolate 31% [sugar, cocoa mass, cocoa butter, emulsifier (soy lecithin), flavor], sugar, rye flour, color (caramel), spices, raising agent (ammonium carbonates), aroma, salt, acidity regulator (citric acid)	wheat flour, chocolate 31% [sugar, cocoa mass, cocoa butter, emulsifier (soy lecithin), flavor], sugar, rye flour, color (caramel), spices, raising agent (ammonium carbonates), powdered elderberry flower extract 0.31%, aroma, salt, acidity regulator (citric acid)	wheat flour, chocolate 25% [sugar, cocoa mass, cocoa butter, emulsifier (soy lecithin), flavor], filling 19% [sugar, concentrated apple puree, concentrated elderberry juice 0.4%, acidity regulator (citric acid), aroma, preservative (potassium sorbate), gelling agent (pectins)], sugar, rye flour, color (caramel), spices, raising agent (ammonium carbonates), powdered elderberry flower extract 0.25%, aroma, salt, acidity regulator (citric acid)

## Data Availability

Not applicable.

## Supplementary Materials

The following supporting information can be downloaded at: https://www.mdpi.com/article/10.3390/ijms24021493/s1.
