# Supplementary material for: Effect of Enriching Gingerbread Cookies with Elder (Sambucus nigra L.) Products on Their Phenolic Composition, Antioxidant and Anti-Glycation Properties, and Sensory Acceptance
Source: Int J Mol Sci. 2023 Jan 12;24(2):1493. doi: 10.3390/ijms24021493 (PMC9865192; doi:10.3390/ijms24021493)
Supplement: Supplementary file 1 [file ijms-24-01493-s001.zip › ijms-2137694-supplementary.pdf]

Table S1. The MS data, parameters of calibration curve, and LOD and LOQ of the analyzed phenolic acids and flavonoids.

No	Compounds	R_t (min)	$[M]^-$ (m/z)	λ_{max} (nm)	a	R^2	LOD (µg/g)	LOQ (µg/g)
				**Phenolic Acids**				
1	*p*-Coumaric acid	2.27	163	309	3E-8	0.999	0.019	0.057
2	*m*-Hydroxybenzoic acid	2.69	137	255	2E-7	0.998	0.013	0.039
3	Caffeic acid	4.56	179	323	1E-8	0.999	0.025	0.076
4	*p*-Hydroxybenzoic acid	4.73	137	255	2E-7	0.999	0.011	0.033
5	Ferulic acid	5.04	193	322	3E-8	1.000	0.012	0.036
6	Benzoic acid	5.18	121	228/272	1E-7	0.999	0.015	0.046
				**Flavonoids**				
7	Myricetin-3-*O*-rutinoside*	4.42	625	357				
8	Quercetin-*O*-hexosyl-*O*-hexoside*	4.53	625	350				
9	Epicatechin	4.58	289	319	6E-8	0.998	0.025	0.076
10	Quercetin-3-*O*-vicianoside*	4.61	595	351				
11	Kaempferol-3-*O*-rutinoside#	4.80	593	351				
12	Quercetin-*O*-pentosyl-hexoside*	4.81	595	354				
13	Quercetin-3-*O*-glucoside*	4.82	463	356				
14	Isorhamnetin-3-*O*-rutinoside*	4.82	623	349				
15	Quercetin-dihexoside*	4.83	625	352				
16	Isorhamnetin-3-*O*-glucoside*	4.97	477	355				
17	Myricetin-3-*O*-glucoside*	5.00	479	354				
18	Quercetin	5.73	301	255, 355	1E-8	1.000	0.022	0.066
19	Apigenin	5.99	269	267, 336	1E-8	0.999	0.010	0.030
20	Naringenin	5.97	271	288	3E-8	0.999	0.015	0.045
21	Kaempferol	6.12	285	319	3E-8	0.998	0.013	0.040

Abbreviations: R_t – retention time; [M]- (m/z) – parent ion; a – calibration slope; R^2 – coefficient of determination; LOD – limit of detection; LOQ – limit of quantification; * – analytical parameters for quercetin were used; # – analytical parameters for kaempferol were used.
